# Unexplained Left Ventricular Hypertrophy Diagnosed as a Cardiac Variant of Late-Onset Fabry Disease: A Case Report

**DOI:** 10.3390/jcdd10090389

**Published:** 2023-09-10

**Authors:** Maomao Zhao, Xiaowei Niu, Lu Bai, Yinchang Zhang, Ting Wang, Yongling Wa, Junchu Wei, Kang Dong, Xin Zhang, Ming Bai

**Affiliations:** 1 Department of Cardiology, First Hospital of Lanzhou University, Lanzhou University, Lanzhou 730030, China; zhaomm2002@163.com (M.Z.); ldyy_niuxw@lzu.edu.cn (X.N.); bl10062023@163.com (L.B.); wangting20210901@163.com (T.W.); a904518994@163.com (Y.W.); eeeegzxc@163.com (J.W.); 15965737119@163.com (K.D.); aszhangxin0705@163.com (X.Z.); 2 Department of Cardiac Surgery, Lanzhou University Second Hospital, Lanzhou University, Lanzhou 730030, China; zychdx@163.com

**Keywords:** left ventricular hypertrophy, Fabry disease, early diagnosis, diastolic dysfunction

## Abstract

The cardiac variant of Fabry disease (FD) has high rates of missed diagnosis and misdiagnosis due to the lack of systemic symptoms. Here, we report a case of a 68-year-old female with delayed-onset FD presenting as concentric left ventricular hypertrophy (LVH) with right bundle branch block, atrial fibrillation, and diastolic dysfunction, which was first presented with coronary artery spasm. Early cardiac-specific signs are crucial for diagnosing this disease due to the lack of extracardiac indications and the late onset of symptoms. This case raises a new red flag that patients with unexplained LVH and its atypical electrocardiographic (ECG) manifestations accompanied by diastolic dysfunction should be considered for FD. We also recommend further refinement of examinations associated with Fabry disease, which will contribute to the early diagnosis and treatment of the disease.

## 1. Introduction

**Figure 1:**
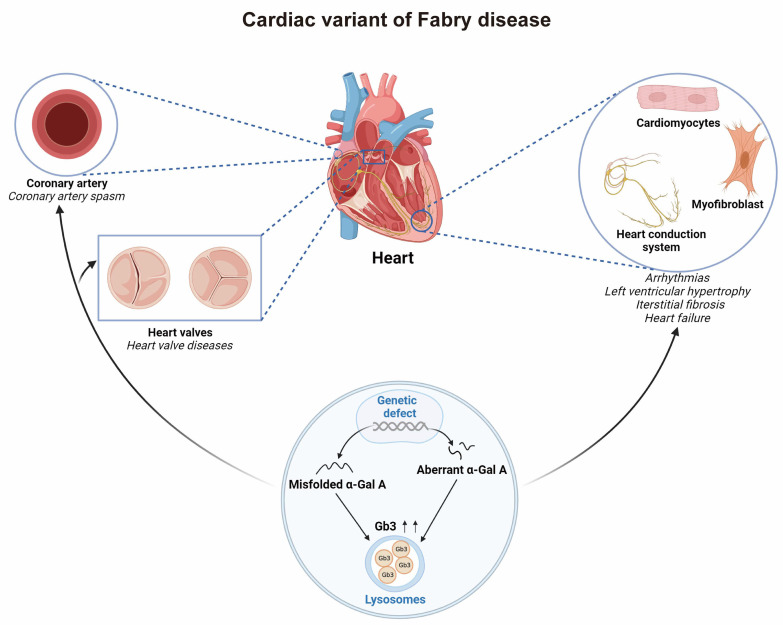
Pathophysiology and relevant mechanisms of cardiac variant of Fabry disease (FD) as a myocardial storage disease. α-Gal A: α-galactosidase A; Gb3: globotriaosylceramide. Figure created using BioRender.com.

Fabry disease (FD) is a rare X-linked inherited lysosomal storage disorder induced by alpha-galactosidase deficiency, resulting in the accumulation of globotriasylceramide (Gb3) in pathological tissues, which can involve multiple organs, with cardiac involvement being the primary cause of mortality in patients with FD [1]. As Gb3 levels rise, cardiomyocytes develop hypertrophy, inflammatory infiltrates, and interstitial fibrosis, and lesions can affect cardiomyocytes, the myocardial interstitium, the conduction system, valves, and blood vessels [2,3]. These conditions ultimately lead to arrhythmias, left ventricular hypertrophy, valvular abnormalities, myocardial fibrosis, and heart failure (Figure 1). Consequently, the early detection of FD prior to irreversible organ injury and the prompt initiation of specific treatments are crucial. Due to the atypical symptoms and lack of systemic involvement of the cardiac variant of late-onset FD, its early diagnosis is typically delayed, which is detrimental to the early treatment and prognosis of the disease.

Here, we present the case of a 68-year-old woman diagnosed with the cardiac variant of FD who exhibited complex clinical manifestations of the cardiovascular system but no extracardiac indications. Initially, the patient was diagnosed with hypertrophic cardiomyopathy (HCM), but FD was suspected based on atypical LVH electrocardiographic (ECG) manifestations. Subsequently, genetic testing and endomyocardial biopsies confirmed the diagnosis.

## 2. Case Presentation

**Figure 2:**
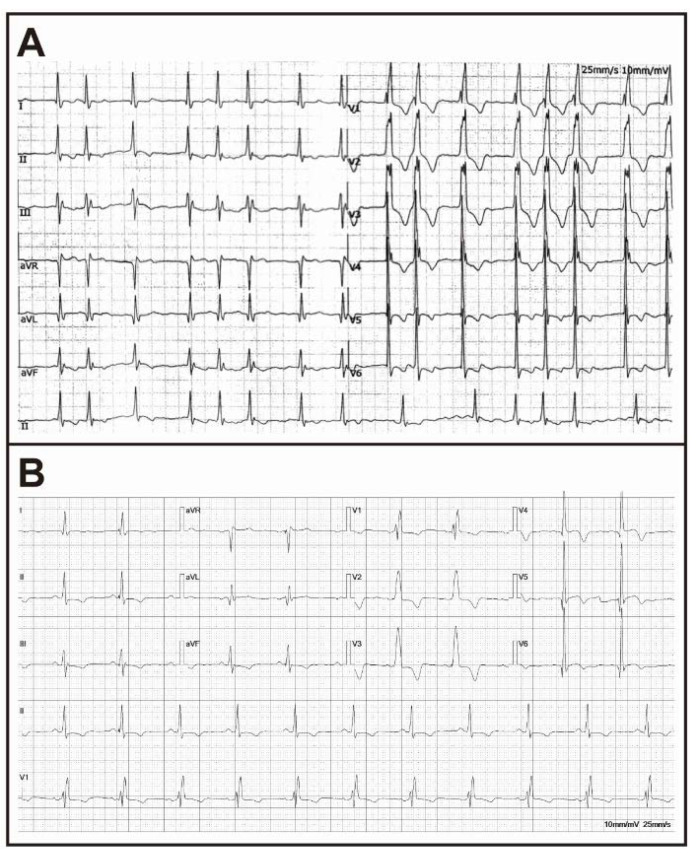
(**A**) A 12-lead ECG 2 years ago showing atrial fibrillation and atypical right bundle branch block. (**B**) This 12-lead ECG shows atypical right bundle branch block, T-wave inversion, and lower wall ST-segment depression.

**Figure 3:**
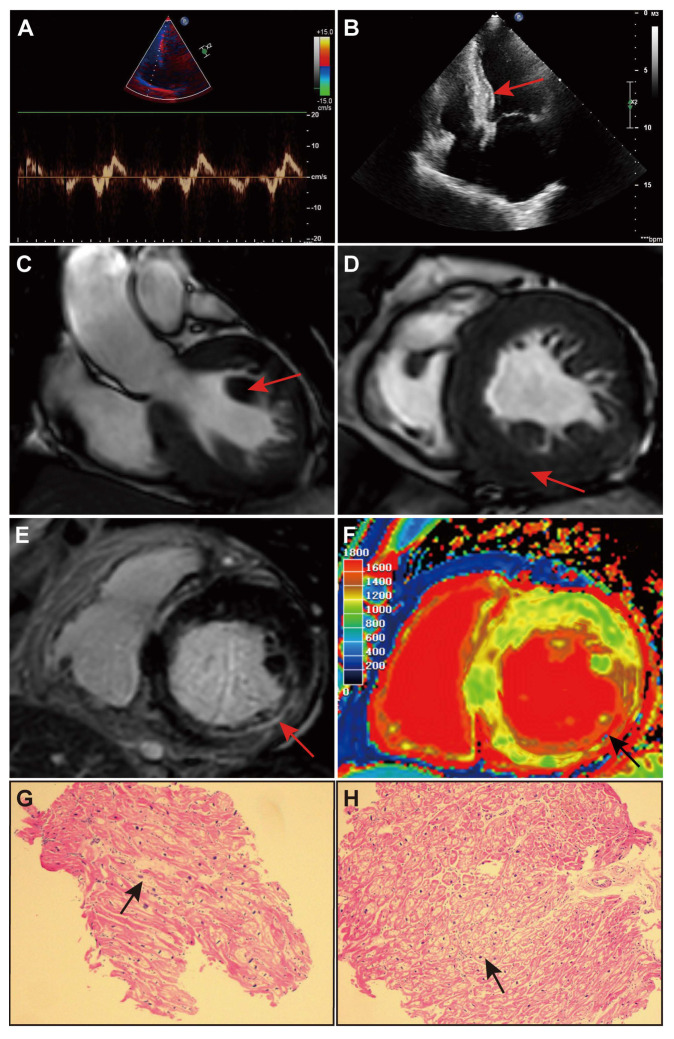
(**A**) TTE for the patient shows decreased left ventricular diastolic function. (**B**) TTE shows left ventricular hypertrophy, especially in the middle of the interventricular septum (red arrow). (**C**) CMR shows papillary muscle hypertrophy (red arrow). (**D**) CMR shows concentric LVH in the patient. (**E**) Late gadolinium enhancement shows limited areas of fibrosis at the level of the septal, inferior, and inferior lateral myocardium (areas of white with red arrow). (**F**) A CMR T1 color map demonstrating elevated T1 signal (areas of red with black arrow). The black arrow referred to the area of increased T1 that corresponds with the area of late gadolinium enhancement. (**G**,**H**) Endocardial biopsy shows most cardiomyocytes are vacuolated (black arrow).

A 68-year-old woman presented with bilateral lower extremities edema and intermittent palpitations to our cardiology department. Laboratory tests showed troponin I (TnI) of 0.14 ng/mL, creatine kinase isoenzyme (CKMB) of 22 ng/mL, and N-terminal pro-B-type natriuretic peptide (NT-proBNP) of 5800 pg/mL. A 12-lead electrocardiogram indicated right bundle branch block, left ventricular hypertrophy, T-wave inversion, and ST-segment depression in the inferior wall (Figure 2B). Transthoracic echocardiography (TTE) showed a thickening of all the walls of the left ventricle, particularly in the mid portion of the interventricular septum, with a maximum thickness of 18 mm, a normal left ventricular ejection fraction, moderate mitral regurgitation, and a markedly enlarged internal diameter of the left atrium and an enlarged internal diameter of the right atrium (Figure 3A,B). Based on these findings, hypertrophic cardiomyopathy was suspected, and the patient was given symptomatic treatment with rivaroxaban tablets (15 mg/d), metoprolol succinate (23.75 mg/d), rosuvastatin tablets (10 mg/d), and spironolactone tablets (20 mg/d). The patient’s bilateral lower limb edema subsided, but they continued to experience intermittent manifestations of palpitation (Table 1).

Four years ago, the patient presented to the local hospital with persistent chest pain. An ECG indicated a complete right bundle branch block with ST-T changes. Laboratory tests showed a TnI of 0.09 ng/mL and a CKMB of 9.9 ng/mL. The patient was diagnosed with coronary artery spasm after coronary angiography revealed no organic stenosis. Therefore, the patient received a dose of aspirin (100 mg/d), ticagrelor (180 mg/d), rosuvastatin tablets (10 mg/d), and nicorandil tablets (15 mg/d).

Two years ago, the patient was revisited at the local hospital for persistent chest pain during sleep. The patient’s ECG suggested atrial fibrillation, complete right bundle branch block, and left ventricular high voltage (Figure 2A). Laboratory tests showed TnI of 0.13 ng/mL, CKMB of 25 ng/mL, and myoglobin of 114 ng/mL. Based on these results, the patient was diagnosed with coronary artery spasm and atrial fibrillation. After receiving rivaroxaban tablets (15 mg/d), metoprolol succinate (23.75 mg/d), rosuvastatin tablets (10 mg/d), and diltiazem hydrochloride tablets (90 mg), the patient experienced no further chest pain episodes.

To further identify the cause of the patient’s arrhythmias and establish a definitive diagnosis, we first performed a cardiac magnetic resonance imaging (MRI) scan, which revealed elevated T1 values, papillary muscle hypertrophy, and concentric LVH (Figure 3C–F). Late gadolinium enhancement showed limited areas of fibrosis at the level of the septal, inferior, and inferior lateral myocardium (Figure 3E), consistent with the CMR characterization of FD. Physicians recommended an endomyocardial biopsy and genetic detection. Subsequently, we took the left intraventricular endomyocardial tissue for biopsy, and light microscopy showed vacuolar degeneration of the majority of cardiomyocytes, increased volume of some cardiomyocytes, and interstitial fibrotic tissue hyperplasia with a small amount of lymphocytic infiltration, which morphologically supported FD (Figure 3G,H).

In addition, we examined the patient’s blood samples, which demonstrated substantially low (<1) α-galactosidase A (α-Gal A) activity, indicating α-Gal A deficiency. Finally, we performed genetic testing on the patient, which revealed a heterozygous variant of p.Arg301Gln (c.902G > C) encoding exon 6 of the GLA gene (NM_000169) (Table 2). Based on the findings of all the aforementioned tests, the patient was ultimately diagnosed with the cardiac variant of delayed-onset FD and had further treatment with ERT. The patient received 1 mg/kg of α-galactosidase every two weeks. At the most recent follow-up in 2023, the patient had no chest pain, palpitations, or bilateral lower extremity edema, and his sleep quality had improved over the previous period. These results indicate that this treatment was effective and delayed the progression of FD.

## 3. Discussion

Due to its low incidence, atypical symptoms, and lack of specific biomarkers, FD is susceptible to missed diagnosis and misdiagnosis, which is detrimental to the early diagnosis, treatment, and prognosis of the disease. Studies have shown that the incidence of FD ranges from 1/40,000 to 1/117,000 [4]. FD involving only the heart is even rarer, resulting in a high rate of misdiagnosis and missed diagnosis in the early phases of the disease. It has been indicated that FD-specific treatment should commence immediately since enzyme replacement therapy will not be able to prevent the progression of organ injury and subsequent mortality once the pathologic manifestations of the disease have fully progressed to fibrosis [5]. Therefore, early diagnosis of the disease and timely initiation of specific therapy are essential to halting the progression of FD and improving the prognosis of patients.

This case describes a patient with a cardiac variant of delayed-onset FD, which is exceptional in having a combination of complex cardiovascular system diseases without extracardiac manifestations in the advanced stages of the disease, including coronary artery spasm, atrial fibrillation, atypical right bundle branch block, and heart failure, with right bundle branch block being even more uncommon. In particular, the QRS pattern is not consistent with the classical ECG-LVH criteria (voltage criteria), even though the patient has FD accompanied by LVH confirmed by CMR. We consider that this may be associated with myocardial injury, which affects the sequence of ventricular activation or delays the activation of the right ventricle, allowing the right ventricle to dominate the electric field, which ultimately presents as right bundle branch block [6]. Initially, physicians diagnosed HCM after evaluating the extent of left ventricular wall thickness, atrial fibrillation, and cardiac function. However, the patient had the atypical characteristic of conventional HCM, namely a right bundle branch block, which is uncommon in the ECG presentations of HCM [7]. To a certain extent, this clue suggests that it may be a particular manifestation of LVH. For patients with unique presentations of LVH in combination with diastolic dysfunction, we should suspect FD and refine the specific examination with FD. The results of the enzyme activity assay, CMR, genetic testing, and endomyocardial biopsy eventually confirmed the diagnosis. Furthermore, the patient’s history of coronary artery spasm also corresponded with the typical manifestations of FD. In particular, echocardiography identifies patients with LVH in combination with diastolic dysfunction and contributes to disease progression monitoring. ECG may aid in identifying patients with typical ECG manifestations of FD or atypical ECG manifestations of HCM, and their red flags may assist in the early diagnosis of FD. At the same time, CMR can detect the level of Gb3 accumulation, cellular edema, and interstitial fibrosis, which can contribute to the diagnosis and staging of FD [8]. In this case, the CMR findings showed areas of elevated T1 values corresponding to areas of delayed enhancement (Figure 3E,F), indicating the presence of fibrosis in the interventricular septum, inferior, and inferior lateral myocardium. Likewise, subsequent follow-up results confirmed this, and the patient did not have extensive areas of myocardium that had progressed to a fibrotic condition, thus not limiting the therapeutic effect. Ultimately, a definitive diagnosis of FD depends on the results of an endomyocardial biopsy and genetic testing.

The current specific therapeutic options for FD include ERT and chaperone therapy, which aim to inhibit the intracellular accumulation of Gb3 by supplementing defective endogenous AGAL (α-galactosidase A) or correcting misfolded endogenous AGAL, resulting in improved transport within the lysosome and increased enzyme activity [9]. After a definitive diagnosis of FD, early initiation of specific therapy can slow the progression of FD and enhance the quality of life. Moreover, this case demonstrates that ERT may delay disease progression in patients with partial myocardial fibrosis due to FD, particularly in terms of symptomatic improvement.

In conclusion, due to the cardiac variant of FD lacking extracardiac manifestations, a delay typically exists between the onset of symptoms and the definitive diagnosis of FD. Therefore, we should suspect FD in patients with unexplained LVH with its atypical ECG manifestations and combined diastolic dysfunction. Meanwhile, refining investigations associated with FD contributes to a definitive early diagnosis of FD and treatments, as well as providing benefits for the prognosis of the patient.

## Figures and Tables

**Table 1:** Clinical history.

Year	Age	Symptoms	Evaluation	Main Diagnosis	Management
2019	64	Chest pain	**ECG**: Atypical right bundle branch block **CAG**: Negative **TTE**: Reduced rate of contraction and thickening of the lower left ventricular wall LVEF = 62%	Acute myocardial infarction and coronary artery spasm	AMI drugs, outpatient follow-up
2021	66	Chest pain and palpitation	**ECG**: Atrial fibrillation and atypical right bundle branch block **TTE**: Left ventricular hypertrophy and LV diastolic dysfunction LVEF = 59%	Acute myocardial infarction, coronary artery spasm, and atrial fibrillation	AF drugs, NDHP-CCB, and outpatient follow-up
2023	68	Palpitation and edema of both lower limbs	**ECG**: Atypical right bundle branch block, atrial fibrillation, and T-wave inversion **TTE**: LVH, LV diastolic dysfunction, enlarged left atrial and right atrial internal diameters LVEF = 63% **CMR**: Hypertrophic cardiomyopathy with diffuse lipid infiltration of the myocardium	Fabry disease, atrial fibrillation, and diastolic heart failure	ERT, AF drugs, HF drugs, and outpatient follow-up

ECG: Electrocardiogram, TTE: Transthoracic echocardiogram, CAG: coronary angiogram, CMR: cardiac magnetic resonance, LVH: left ventricular hypertrophy, LV: left ventricular, AMI: acute myocardial infarction, AF: atrial fibrillation, HF: heart failure, NDHP-CCB: nondihydropyridine- calcium channel blocker, ERT: enzyme replacement therapy.

**Table 2:** Examination related to Fabray disease.

Examination	Results	Reference Value
α-Gal A activity	<1 nmol/1 h/mg	N > 24.70 (nmol/1 h/mg)
Endomyocardial biopsy	Most cardiomyocytes are vacuolated	Compatible with FD
Genetic sequencing	p.Arg301Gln (c.902G > C) heterozygous variant encoding exon 6 of the GLA gene (NM_000169)	Compatible with FD

α-Gal A: α-galactosidase A, N: normal value, FD: Fabry disease.

## Data Availability

Not applicable.
